# Rotational Detection of the Missing Conformers of 2‑Chloropropionic Acid

**DOI:** 10.1021/acs.jpca.5c07699

**Published:** 2026-01-15

**Authors:** Fufei Sun, Assimo Maris, Luca Evangelisti, Wentao Song, Sonia Melandri, J. Ricardo Morán, Camilla Calabrese, Alberto Lesarri, Jens-Uwe Grabow

**Affiliations:** † Department of Chemistry “Giacomo Ciamician”, 9296 University of Bologna, Via Gobetti 85, 40129 Bologna, Italy; ‡ Departamento de Química Física y Química Inorgánica, 16782 Facultad de Ciencias—I.U. CINQUIMA, Paseo de Belén, 7, 47011 Valladolid, Spain; § Institut für Physikalische Chemie und Elektrochemie, 26555 Gottfried-Wilhelm-Leibniz-Universität Hannover, Callinstrasse 3A, 30167 Hannover, Germany

## Abstract

The conformational space of 2-chloropropionic acid was reinvestigated using millimeter-wave and chirp-polarization Fourier transform microwave spectroscopies under isolated conditions in supersonic expansions. Besides the global minimum (carbonyl oxygen eclipsed with the methyl group), two additional conformers were identified: one with the carbonyl eclipsed relative to the α-C–H bond, and another stabilized by an intramolecular OH···Cl hydrogen bond. The latter interaction is evidenced by a decrease in |χ_*zz*_|: the chlorine nuclear quadrupole coupling constant associated with the principal axis lying along the C–Cl bond and an increase in the asymmetry parameter η = (χ_*xx*_ – χ_*yy*_)/χ_*zz*_ of the chlorine quadrupole coupling tensor. Rotational constants, geometries, and relative energies were determined and compared with quantum-chemical calculations, revealing larger deviations for conformers with intramolecular interactions. Calculations also show that chlorination markedly modifies the conformational landscape relative to propionic acid, affecting both minima and interconversion barriers. The accurate reproduction of the electronic properties encoded in the determined complete nuclear quadrupole coupling tensors requires the inclusion of relativistic approaches.

## Graphical abstract

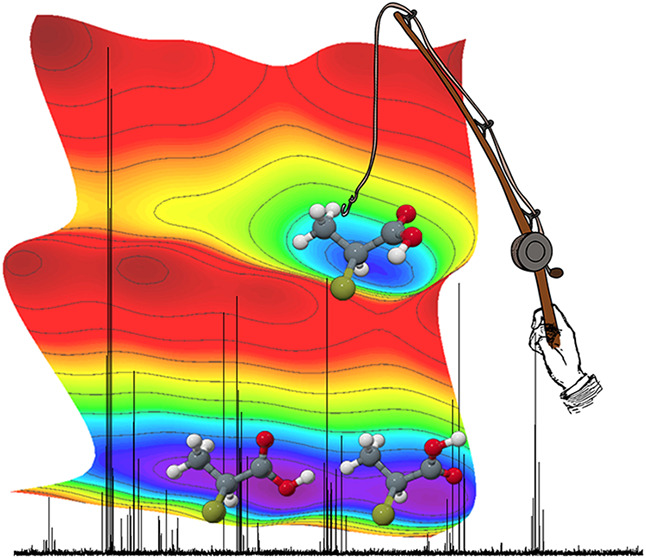

## Introduction

Halogenated carboxylic acids (XCAs) represent an important class of organic compounds due to their significance in both synthetic chemistry and environmental science.^1^ They are widely used as refrigerants, flame retardants, solvents, and pharmaceuticals^2^ but their dispersion in the environment poses potential risks to agricultural soil and groundwater.^3^ Once these compounds enter the human food chain or drinking water, they can bioaccumulate in organisms, thereby threatening human health. A retrospective in silico screening of analytical data identified several XCAs in drinking water as disinfection byproducts.^4^ The ability of XCAs to pass through conventional drinking water purification processes is largely attributed to their polarity, which governs their solubility and adsorption in water, and consequently influences their mobility in aquatic systems.^3^ Since molecular polarity is a global property that relates to molecular structure, investigating the structures of such compounds is crucial for understanding and predicting their mobility in water. In particular, it has been pointed out that for flexible molecules a proper modeling of the conformational space is essential for the correct prediction of physical variables both in the pure phase and in solution.^5^

High-resolution spectroscopic methods on supersonic expansions were shown to be very successful experimental techniques for the study of complex conformational spaces of molecules and molecular complexes, allowing the determination of the molecular inherent structural preferences and their changes upon complexation.^6^ In particular, pure rotational spectroscopy in the gas phase enables precise determination of molecular structures,^7^ conformational landscapes,^8^ and internal dynamics^9^ as well as intra- and intermolecular interactions unbiased from environmental constraints.^10^

Among the smaller XCAs, chloroproprionic acids have been the subject of investigation. Our recent rotational spectroscopy study on 3-chloropropionic (3ClPA),^11^ the β-chloro compound, shows a complex conformational space with several minima in consequence of the hindered torsion around the single bonds. The most stable are conformers with the carboxylic group in the *zusammen* (*Z-*COOH) arrangement, followed by the *entgegen* (*E-*COOH) conformers, the lowest of which with a calculated relative energy of more than 17 kJ mol^–1^ (Δ*E*_0_, B3LYP-D3­(BJ)/def2-TZVP) with respect to the global minimum. The study identified the rotational spectra originating from the three most stable *Z*-COOH conformers demonstrating the use of the heavier carrier gas Ar to reduce the number of populated conformations due to more efficient collisional relaxation compared to He as expansion medium. The derived geometries, spectroscopic parameters and energy ordering can be directly used to benchmark the results of B3LYP-D3­(BJ)/def2-TZVP, MP2/aug-cc-pVTZ, and quantum chemical predictions corrected for relativistic effects.

For the α-chloro compound 2-chloropropionic acid (2ClPA) investigated here, a similar complex conformational space is expected with differences from the β-chloro compound related to the chlorine atom’s position being closer to the carboxylic group. A previous gas phase conformational study reported the detection of only one conformation, i.e. the global minimum where the carboxylic group is in the *Z*-COOH arrangement with the methyl and carbonyl groups being eclipsed.^12^ A subsequent study in solution^13^ focused on the strong and weak hydrogen bonds (HBs) influencing the Vibrational Circular Dichroism (VCD) spectra, finding that the complexity and intensity of the VCD spectra are especially affected by the choice of solvent via HB formation. The experimental study, paired with high level quantum mechanical calculations, showed the presence of three conformers for the monomer while several hydrogen bonded complexes are formed in solutions, highlighting that solvation and intermolecular interactions can mask intrinsic molecular conformations, underlining the importance of gas phase studies to reveal the intrinsic molecular preferences.

In both studies^12,13^ two higher-energy conformations of 2ClPA adopting *Z*-COOH and *E*-COOH arrangement of the carboxyl group were predicted at relative small energies of 1.41 kJ mol^–1^ and 5.01 kJ (Δ*E*_0_/kJ mol^–1^ B3LYP-D3­(BJ)/def2-TZVP) respectively, with respect to the global minimum. These conformations were not observed in the gas phase study even if expected to be energetically accessible. The authors rationalized this by collisional relaxation of the higher energy conformations during supersonic expansion.

Prompted by the results obtained on 3ClPA and the reported theoretical calculations on 2ClPA which pointed to a complex conformational surface, the reinvestigation of 2ClPA in a He expansion using free-jet Stark-modulation millimeter-wave (FJ-AMMW) spectroscopy and the more sensitive chirp-polarization Fourier transform microwave (CP-FTMW) spectroscopy technique was deemed extremely interesting to refine the conformational landscape information in order to explore the intramolecular interactions involved in the stabilization of its conformers. Highlighting the impact of the substituents’ position on the molecules’ structural and electronic properties is the ultimate aim of the present paper.

## Experimental and Computational Methods

The rotational spectra of 2ClPA were initially measured using a Stark-modulated FJ-AMMW spectrometer operating in the 59.6–78.3 GHz frequency range, which has been previously described.^14–16^ 2ClPA is a transparent liquid at room temperature exhibiting a vapor pressure of 100 Pa (293 K) and a boiling temperature of 443–463 K. Without further purification, the sample was heated to 323 K under a flow of He. The resulting gas mixture was continuously expanded from a stagnation pressure of 32 kPa into a vacuum chamber maintained at a background pressure of 0.2 Pa through a nozzle with a diameter of 0.3 mm. The estimated accuracy of the frequency measurements is 50 kHz, allowing the resolution of lines separated by >300 kHz.

The spectra in the 2–8 GHz frequency range were collected using a CP-FTMW spectrometer also equipped with a supersonic jet source. Here, a He/Ar mixture was flown over the sample, also heated to 333 K and pulse-expanded through a solenoid valve into the chamber. The backing pressure of the inert gas mixture was kept at 203 kPa. The direct-digital CP-FTMW instrument uses an arbitrary-waveform generator (25 GSamples/sec) digital source, followed by amplification with a traveling-wave tube (250 W). Sets of eight subsequent chirp-polarization experiments were used on each gas pulse. Horn antennae, oriented perpendicularly to the jet, were used for transmission of the excitation radiation as well as for reception of the molecular response. The estimated accuracy of the frequency measurements is 10 kHz.

The molecular structures were predicted at the B3LYP-D3­(BJ)/def2-TZVP and MP2/aug-cc-pVTZ levels of theory using the Gaussian software package (G16, revision C.01)^17^ with harmonic frequency calculations confirming the determined structures to be real minima. The MP2 structures were used to estimate the nuclear quadrupole coupling constants (NQCC) of the chlorine nucleus using the Douglas-Kroll-Hess second-order scalar relativistic core Hamiltonian^18^ in the point nuclear approximation with the recontracted aug-cc-pVTZ-DK^19,20^ basis set (freely available at the Basis Set Exchange Database^21^). We indicate this method with the acronym MP2//DK. These calculation levels behaved satisfactorily in our previous studies of similar molecules, e.g. 3ClPA^11^ and aryl halides.^22^

Minimum energy conformational pathways were explored at the B3LYP-D3­(BJ)/def2-TZVP level through relaxed scans using a 20 or 10° step in the full cycle of rotation. To quantify the energy related to HBs, the topology of the theoretical electron densities were analyzed with the Multiwfn program^23^ based on the Atoms in Molecules Theory (AIM).^24^ Complementary information was achieved from visualizing the noncovalent interactions (NCI) with the NCI method,^25^ which considers the distribution of both the electron density (ρ), and its gradient (σ) as well as its second derivatives matrix (λ_1_, λ_2_, λ_3_). A comprehensive picture can be drawn using different plots of these quantities. Color coded isosurfaces visible in the NCI plots represent the area for attractive or repulsive interactions.

## Results and Discussion

### Potential Energy Surface and Rotational Spectrum

With the terminal methyl group assuming *C*_3*v*_ symmetry, only the skeletal CC-C­(OH) (τ_1_) and HO-C=O (τ_2_) torsion angles determine the conformations of 2ClPA as depicted in Figure 1. The energy scan represented in Figure 2 uses these two dihedral angles with the structures of the three energy minima optimized subsequently. In the two most stable rotamers the carboxylic group is planar with the hydroxylic hydrogen atom and carboxylic group being on the same side: *zusammen* (*Z*) configuration while in the third rotamer the carboxylic group and hydroxylic hydrogen are on opposite sides: *entgegen* (*E*) configuration, allowing the interaction of the chlorine atom and the hydroxylic hydrogen. The three conformations are shown in Figure 1, labeled with successive numbers according to their energy order. Complete sets of spectroscopic constants are given in Table 1. It must be noted that, since the α carbon atom is chiral, each conformation possesses two enantiomeric forms, but because of their spectroscopic equivalence, we only depict the S(−) forms.

**Figure 1:**
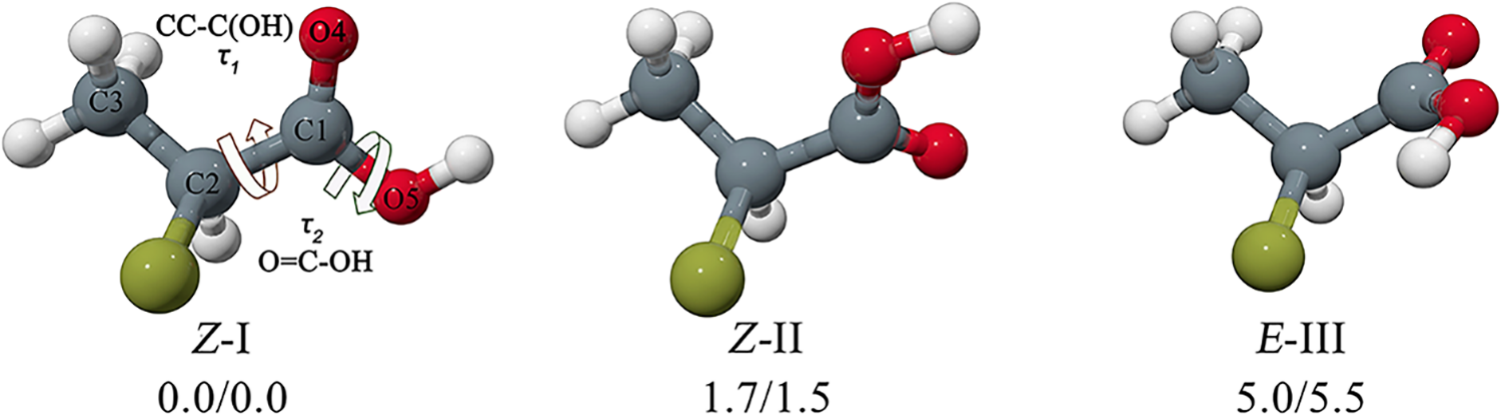
Geometric structures and zero-point corrected relative energies (Δ*E*_0_/kJ mol^–1^ B3LYP-D3­(BJ)/def2-TZVP/MP2/aug-cc-pVTZ) of the 3 most stable conformers of 2ClPA. Only the S enantiomers are shown.

**Figure 2:**
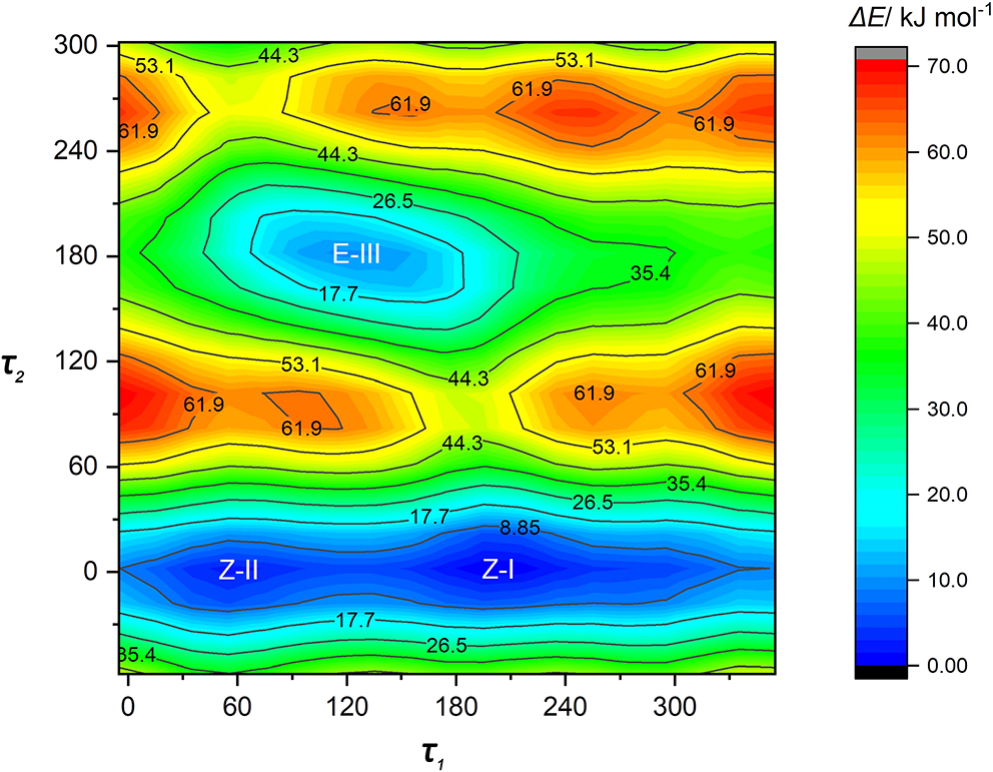
2D potential energies (relative electronic energies in kJ mol^–1^) of 2ClPA as a function of the torsional angles CC-C­(OH) (τ_1_) and HO-C=O (τ_2_). Structurally relaxed scans of the dihedral angles were performed at the B3LYP-D3­(BJ)/def2-TZVP level using 20° steps in the full cycle of rotation.

**Table 1:** Theoretically Predicted Spectroscopic Constants for the Three Observed Conformers (^35^Cl Isotopologue) of 2ClPA (B3LYP-D3­(BJ)/def2-TZVP and MP2/aug-cc-pVTZ)

	*Z*-I		*Z*-II		*E*-III	
parameter^a^	B3LYP	MP2	B3LYP	MP2	B3LYP	MP2
τ_1_/°	203.1	200.8	54.6	56.5	114.7	110.5
τ_2_/°	2.6	2.5	–2.0	–1.8	–178.0	–177.2
*E*_*e*_ /a.u.	–728.152924	–727.065841	–728.152263	–727.065255	–728.151017	–727.063718
*E*_0_/a.u.	–728.071513	–726.983632	–728.070841	–726.983061	–728.069692	–726.981541
Δ*E*_*e*_ /kJ mol^–1^	0.00	0.00	1.76	1.54	4.78	5.57
Δ*E*_0_/kJ mol^–1^	0.00	0.00	1.74	1.50	5.01	5.49
*N*_i_/*N*_*I*_ (333 K)	1	1	0.53	0.58	0.16	0.14
*N*_i_/*N*_*I*_ (323 K)	1	1	0.52	0.57	0.15	0.13
*A*_e_/MHz	3877.39	3902.05	4013.21	4071.17	4279.95	4325.46
*B*_e_/MHz	2297.15	2336.53	2105.80	2117.47	2264.16	2271.71
*C*_e_/MHz	1743.69	1757.61	1844.94	1870.26	1628.05	1659.20
*D*_*J*_/kHz	0.87	0.91	1.30	1.66	0.53	0.52
*D*_*JK*_/kHz	1.36	2.02	–0.28	–0.21	–0.01	0.08
*D*_*K*_/kHz	–0.83	–1.56	2.90	4.07	1.02	0.99
*d*_1_/kHz	–0.28	–0.31	–0.24	–0.24	–0.15	–0.13
*d*_2_/kHz	–0.16	–0.20	0.29	0.47	–0.15	–0.15
3/2*χ*_*aa*_/MHz	–29.07	–24.39	–37.80	–33.65	–58.42	–51.79
(χ_bb_ – *χ*_cc_)/4/MHz	–3.01	–2.11	–7.87	–7.40	–8.29	–7.40
χ_ab_/MHz	–45.80	–39.96	–51.21	–45.95	46.72	42.05
χ_ac_/MHz	–37.02	–33.63	–27.73	–23.54	11.22	9.71
χ_bc_/MHz	–30.61	–27.64	–23.12	–19.63	–10.92	–9.55
|μ_a_|/D	0.62	0.56	0.31	0.26	2.76	2.78
|μ_b_|/D	0.24	0.26	1.82	1.71	0.96	1.01
|μ_c_|/D	1.78	1.67	0.84	0.82	0.23	0.27
μ_tot_/D	1.90	1.79	2.03	1.91	2.93	2.97
*P*_*aa*_/uÅ^2^	189.74	187.16	194.00	192.38	207.77	205.11
*P*_bb_/uÅ^2^	100.08	100.38	79.93	77.84	102.65	99.48
*P*_cc_/uÅ^2^	30.26	29.14	46.00	46.29	15.43	17.36
κ	–0.48	–0.46	–0.76	–0.78	–0.52	–0.54

a τ_1_ and τ_2_ are the CC-C­(OH) and HO-C=O torsional angles; *E*_e_ is the absolute electronic energy and *E*_0_ is the absolute zero-point corrected energy; Δ*E*_e_ is the relative electronic energy and Δ*E*_0_ is the relative zero-point corrected energies; *N*_i_/*N*_0_ is the population ratio with respect to the global minimum conformer calculated using the relative zero-point corrected energy; *A*_e_, *B*_e_, and *C*_e_ are the equilibrium rotational constants. *D*_J_, *D*_JK_, *D*_K_, *d*_1_, and *d*_2_ are the quartic centrifugal distortion constants of Watson’s *S*-reduced semirigid rotor Hamiltonian in its *I* ^r^-representation. χ_aa_, χ_bb_, and χ_cc_ are the nuclear quadrupole coupling constants in the principal inertial axis system representation. μ_a_, μ_b_, μ_c_ are the electric dipole moment components. *P*_gg_ (*g* = *a*, *b* or *c*) are the planar moments of inertia, i.e. *P*_cc_ = (*I*_aa_ + *I*_bb_ – *I*_cc_)/2. κ = (2*B* – *A* – *C*)/(*A* – *C*) is Ray’s asymmetry parameter.

As described in the introduction, among the possible stable conformations, only the global minimum was observed in a previous rotational spectroscopy study. The authors explain that the higher energy conformer, i.e. *Z*-II. may be depleted due to collisional relaxation processes to the most stable one, while the *E-*III conformer was deemed too high in energy for a population showing a spectrum. However, according to our calculations, the *Z*-II to *Z*-I interconversion barrier is large enough (4.5 kJ mol^–1^) not to be overcome in a He expansion (see Figure 2). Indeed, new measurements performed with the FJ-AMMW spectrometer using He as carrier gas allowed us to detect several new μ_b_-R-type transition lines (up to *J* = 13 and *K*_a_ = 8) for both the parent and the weaker ^37^Cl isotopologue (24.24% in natural abundance). The newly determined spectroscopic constants for this second species (II) are reported in Table 2. Indeed, comparison of the determined rotational constants with the calculated parameters of Table 1 allows the assignment to conformer *Z*-II.

**Table 2:** Experimentally Determined Spectroscopic Constants of the Three Observed Conformers (^35^Cl and ^37^Cl Isotopologues) of 2ClPA

parameters^a^	^35^Cl–I	^37^Cl–I	^35^Cl–II	^37^Cl–II	^35^Cl–III	^37^Cl–III
*A*_0_/MHz	3895.3364(4)^b^	3868.6410(6)	4059.49(1)	4033.311(3)	4279.565(3)	4267.629(2)
*B*_0_/MHz	2345.5558(2)	2295.7225(7)	2109.56(3)	2067.8(2)	2287.532(1)	2232.285(7)
*C*_0_/MHz	1734.0079(4)	1702.8730(7)	1859.62(2)	1822.5(2)	1637.7161(9)	1607.7228(7)
*D*_J_/kHz	0.81(1)	0.77(3)	1.42(2)	[1.42]^c^		
*D*_JK_/kHz	2.59(4)	2.7(1)				
*D*_K_/kHz	–1.94(3)	–2.0(1)	3.3(1)	[3.3]	1.9(8)	[1.9]
*d*_1_/kHz	–0.256(6)	–0.23(2)			–0.16(2)	[-0.16]
*d*_2_/kHz	–0.216(2)	–0.20(2)	0.44(2)	[0.44]	–0.205(7)	[-0.205]
1.5χ_aa_/MHz	–26.552(8)	–23.501(8)	–35.8(7)	–31.9(8)	–54.53(1)	–44.24(2)
0.25(χ_bb_ – χ_cc_)/MHz	–2.423(2)	–1.710(3)	–8.4(9)	–6.59*^d^	–7.766(4)	–5.882(5)
χ_ab_/MHz	42.8(6)	33.1(8)			43.6(7)	32.9(8)
χ_ac_/MHz	–36.2(8)	–28.7(9)			11(1)	8.67*
χ_bc_/MHz	28.8(5)	22.1(6)			–11.5(9)	–9(1)
σ/kHz	10	11	26	24	7	8
*N*	169	92	37	12	60	41
μ_a_/D	Y	Y	N	N	Y	Y
μ_b_/D	Y	Y	Y	Y	Y	Y
μ_c_/D	Y	Y	N	N	N	N
*P*_aa_/uÅ^2^	188.6	193.1	193.4	198.2	205.7	211.2
*P*_bb_/uÅ^2^	102.9	103.6	78.4	79.1	102.9	103.2
*P*_cc_/uÅ^2^	26.9	27.0	46.2	46.2	15.2	15.2
κ	–0.43	–0.45	–0.77	–0.78	–0.51	–0.53

a *A*_0_, *B*_0_, and *C*_0_ are the ground-state rotational constants. *D*_J_, *D*_JK_, *D*_K_, *d*_1_, and *d*_2_ are the quartic centrifugal distortion constants of Watson’s *S*-reduced semirigid rotor Hamiltonian in its *I* ^r^-representation. χ_aa_, χ_bb_, and χ_cc_ are the nuclear quadrupole coupling constants in the principal inertial axis system representation. *N* is the number of quadrupole hyperfine components fitted. σ is the rms deviation of the fit. μ_a_, μ_b_, μ_c_ are the electric dipole moment components, Y or N indicates if the corresponding transition lines have been observed. *P*_gg_ (*g* = *a*, *b* or *c*) are the planar moments of inertia, i.e., *P*_cc_ = (*I*_aa_ + *I*_bb_ – *I*_cc_)/2, κ = (2*B* – *A* – *C*)/(*A* – *C*) is Ray’s asymmetry parameter.

b Standard errors in parentheses in units of the last digit.

c Values in the brackets are fixed to the values of the parent species.

d Values marked with an asterisk are fixed to the scaled parent species values.

The rotational spectrum of 2ClPA was also collected using the more sensitive pulsed-jet CP-FTMW technique in the frequency range 2–8 GHz using an Ar/He supersonic expansion. New transitions for conformer *Z*-I in the lower frequency range were observed allowing for a global fit extending the previously measured set of millimeter-wave transitions.^12^ Initially, no transitions for *Z*-II were observed in the CP-FTMW spectrum, consistent with the conformational relaxation from *Z*-II to *Z*-I in the Ar/He expansion hypothesized previously. However, due to the higher sensitivity of the technique, several lines of a third conformer could be detected. One of the most intense ones is the 2_0,2_ ← 1_0,1_ μ_a_-R-type transition which shows the typical nuclear quadrupole hyperfine structure shown in Figure 3. Following the initial observations, it was possible to measure several μ_a_ and μ_b_ R-type transition lines (up to lower *J* = 4 and *K*_a_ = 2) and analog transitions of the ^37^Cl isotopologue for this conformer which can be identified as *E*-III.

**Figure 3:**
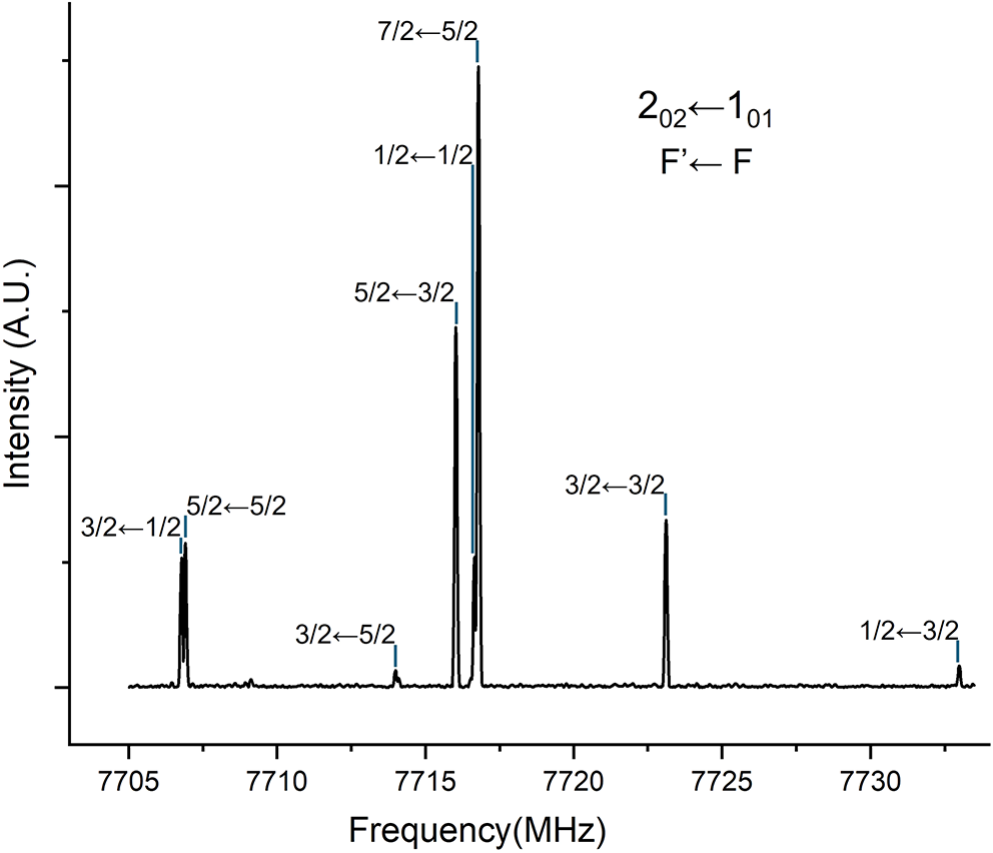
2_0,2_ ← 1_0,1_ transition of the *E-*III conformer (^35^Cl) showing fully resolved chlorine nuclear quadrupole hyperfine structure (quantum numbers F’ ←F are shown). The spectrum was obtained by averaging 27 free induction decays.

All transition frequencies were fitted using the SPFIT code from Pickett’s CALPGM suite of programs.^26^ Watson’s semirigid Hamiltonian was set up using the *S* reduction in its *I* ^r^-representation, supplemented with nuclear quadrupole coupling (NQC) terms.^27^ The number of spectroscopic parameters determined for conformer *Z*-I is the same with respect to the previous rotational spectroscopy study but their precision is slightly increased. The complete NQC tensor could be determined for *Z*-I and *E*-III species except ^37^Cl–III for which only the diagonal terms were obtained while the off-diagonal ones were fixed to the values obtained by rotating the fitted ^35^Cl–III values in the PAS of the ^37^Cl isotopologue and scaled by the corresponding nuclear quadrupole moment values.^28^ The nuclear quadrupole moment of ^35^Cl (*Q* = −81.65(80) mb) is larger than that of ^37^Cl (−64.35(64) mb)^29,30^ making the splitting of the rotational levels caused by ^37^Cl smaller than those of ^35^Cl. All the spectroscopic parameters obtained are shown in Table 2 and all transition lines are listed in the Supporting Information (Tables S1–S6).

### Molecular Structure

The structural parameters reported in Table 1 correspond to the equilibrium structure (i.e., rotational constants *A*_e_, *B*_e_, and *C*_e_) derived from quantum-chemical calculations, representing the vibrationless configuration at the minimum of the potential energy surface (PES). In contrast, the experimental data (Table 2) provide the ground-state rotational constants (*A*_0_, *B*_0_, and *C*_0_), which include the effects of zero-point vibrations. Nevertheless, comparison between experimental and theoretical values allows an assessment of the accuracy of the computational methods in reproducing the molecular geometry. For the rotational constants of *Z*-I and *Z*-II, the MP2/aug-cc-pVTZ method (deviations up to 1.4%) appears more accurate than B3LYP- D3­(BJ)/def2-TZVP (deviations up to 2.1%). For *E*-III, the contrary is observed and B3LYP-D3­(BJ)/def2-TZVP (deviation up to 1%) agrees better than MP2/aug-cc-pVTZ (deviation up to 1.3%). This is consistent with the better prediction agreement of the B3LYP/def2-TZVP method found for the conformations of 3ClPA, which show intramolecular interactions.^11^ Overall, we can see that B3LYP-D3­(BJ)/def2-TZVP underestimates the rotational constants while MP2/aug-cc-pVTZ overestimated them.

Using the experimental rotational constants obtained for the normal species and the monosubstituted ^37^Cl isotopologues, we calculated the experimental *r*_*s*_ coordinates of the chlorine atom using Kraitchmann’s substitution method^31^ with Costain’s error estimation.^32^

This analysis leads to the determination of the principal axis coordinates of the substituted atoms from the change resulting in the principal moments of inertia by the monoisotopic substitution. In this way, we obtained the substitution coordinates of the chlorine atom in the principal inertial axes system of the unsubstituted molecule from the rotational constants of the ^35^Cl and ^37^Cl species. The coordinates are obtained as absolute values, but their signs can be easily reconstructed by comparison with the predictions. The derived chlorine vibrational ground-state substitution coordinates are shown in Table 3 and compared to the predicted equilibrium structure values. In this zeroth-order comparison of the experimental and theoretical values, the DFT method overestimates the distance of the chlorine atom from the center of mass (deviations below 1.5%) while the MP2 method tends to underestimate it (with deviations below 0.5%).

**Table 3:** Experimental Substitution Coordinates (*r*_*s*_) and Theoretical Equilibrium Coordinates (*r*_*e*_, B3LYP-D3­(BJ)/def2-TZVP, MP2/aug-cc-pVTZ) of the Cl Atom in the Principal Axes System of the Normal Species of 2ClPA

*Z*-I	*r*_*s*_	*r*_*e*_/B3LYP	*r*_*e*_/MP2
*a* (Å)	±1.517(1)^a^	–1.5082	–1.5078
*b* (Å)	±0.644(2)	–0.6461	–0.6487
*c* (Å)	±0.253(6)	–0.2664	–0.2673
*Z*-II	*r*_*s*_	*r*_*e*_/B3LYP	*r*_*e*_/MP2
*a* (Å)	±1.556(4)	–1.5624	–1.5512
*b* (Å)	±0.63(1)	–0.6679	–0.665
*c* (Å)	±0.18(3)	–0.0572	–0.0239
*E*-III	*r*_*s*_	*r*_*e*_/B3LYP	*r*_*e*_/MP2
*a* (Å)	±1.6637(9)	1.6790	1.6631
*b* (Å)	±0.409(4)	–0.4026	–0.4008
*c* (Å)	±0.11(1)	0.1123	0.1285

a Standard error in parentheses in units of the last digit using Costain’s error estimation.

Additional structural and electronic information can be derived from the NQC tensor, which is related to the electric field gradient (EFG) tensor at the quadrupole nucleus. Since all off-diagonal elements of the NQC real symmetric tensor are determined for *Z*-I and *E*-III in the inertial principal axis system (I-PAS), the NQCCs in their own principal axis system (EFG-PAS) can be obtained experimentally by diagonalizing the tensor. These values and associated errors were calculated using the QDIAG, available on the PROSPE site.^33^ The diagonal values are reported in Table 4 for ^35^Cl–I and ^35^Cl–III together with their associated theoretical predictions at various levels of theory.

**Table 4:** Experimental and Theoretical NQCCs in the Principal Axis System of the Coupling Tensor (EFG-PAS) and Rotation Angles of Principal Inertial Tensor (I-PAS) Axes (*a*, *b*, *c*) with EFG axis (*z*), and Theoretical Angles between the Principal Inertia Tensor Axis (*a*, *b*, *c*) and Cl–C Bond

2ClPA		Exp	B3LYP^a^	MP2^b^	MP2//DK
^35^Cl–I	χ_*xx*_/MHz	36.6(9)^c^	38.41	34.14	35.78
	χ_*yy*_/MHz	38.1(6)	40.93	36.16	37.94
	χ_*zz*_/ MHz	–74.6(9)	–79.34	–70.30	–73.72
	η^d^	0.02(1)	0.03	0.03	0.03
	|θ_(za)_|^e^/degrees	45.7(1)	45.51	45.98	45.91
	|θ_*(*zb)_|/degrees	123.0(2)	123.57	123.21	123.26
	|θ_(zc)_|/degrees	62.4(3)	63.19	62.23	62.36
	|θ_a_|^f^/degrees		47.1	47.4	
	|θ_b_|/degrees		125.0	124.7	
	|θ_c_|/degrees		63.0	62.2	
^35^Cl–III	χ_*xx*_/MHz	29(1)	31.75	28.58	30.67
	χ_*yy*_/MHz	38.1(8)	39.67	35.26	36.28
	χ_*zz*_/MHz	–67.1(7)	–71.42	–63.83	–66.94
	η	0.13(2)	0.11	0.10	0.08
	|θ_(za)_|/degrees	34.3(2)	34.05	34.18	34.01
	|θ_(zb)_|/degrees	122.9(2)	122.80	123.00	122.85
	|θ_(zc)_|/degrees	98.5(5)	98.14	97.94	97.83
	|θ_a_|/degrees		34.05	34.33	
	|θ_b_|/degrees		122.95	123.3	
	|θ_c_|/degrees		97.65	97.3	

a B3LYP-D3­(BJ)/def2-TZVP.

b MP2/aug-cc-pVTZ.

c Errors in parentheses in units of the last digit.

d η = (χ_*xx*_ – χ_*yy*_)/χ_*zz*_.

e Angles between the electric field gradient *z*-axis and the principal inertial axes (*a*, *b*, *c*).

f Angles between the C–Cl bond axis and the principal inertial axes (*a*, *b*, *c*).

From the structural data reported in this table, in particular from the comparison of the angles between the C–Cl bond axis and the principal inertial axes (*a*, *b*, *c*) and the angles between the electric field gradient *z* axis and the principal inertial axes (*a*, *b*, *c*) which are almost coincident, we can assert that the *z* electric field gradient tensor axis is coincident with the C–Cl bond axis, as expected. In addition, the values of the asymmetry parameter η = (χ_*xx*_ – χ_*yy*_ *)*/χ_*zz*_ are also presented to characterize the symmetry of the electric field gradient of the chlorine atom around the *z* axis. The small η value of ^35^Cl–I corresponds to a nearly cylindrical symmetry of the electric field gradient at the chlorine atom, suggesting an unperturbed C–Cl σ-bond character. However, the η value of ^35^Cl–III is significantly larger, indicating an asymmetric arrangement of the electric field gradient, which may be caused by the Cl···HO intramolecular interaction. Regarding the accuracy of the theoretical predictions in reproducing the NQCCs for the chlorine atom, we can see that B3LYP-D3­(BJ)/def2-TZVP overestimates the values while MP2/aug-cc-pVTZ underestimates them at similar deviation (up to 9%). A better agreement can be found using the MP2//DK method, which accounts for relativistic effects (deviations below 5%).

To further clarify the impact of molecular structure on the NQCCs, we conducted a comparative analysis of the χ_*zz*_ and η values in the 2ClPA conformers and other studied chlorine-containing molecules. As shown in Table 5, we observed that the values of χ_*zz*_ are similar for all molecular systems, while the values of η exhibited significant differences. Notably, the η value of 2ClPA-III is markedly larger than those of the other conformations, indicating an asymmetric electric field distribution around the chlorine atom. In contrast, the η values for the other molecules are close to zero, suggesting that their electric field distributions along χ_*zz*_ are more symmetrical.

**Table 5:** Experimental Comparison of χ_*zz*_ and η Values for Different Conformations of 2-Chloropropionic Acid and Other Molecules

	χ_*zz*_/MHz	η
3ClPA-I	–71.6(3)^11^	0.007(5)
3ClPA-II	–71.3(8)	0.07(2)
3ClPA-III	–71.9(1)	0.03(1)
2ClPA-I	–74.6(9)	0.02(1)
2ClPA-III	–67.1(7)	0.13(2)
HCl	–67.61 ^34^	0
CH_3_Cl	–74.753(2)^35^	0
C_3_H_7_Cl(trans)	–70.68(11)^36^	0.009(2)

### Intramolecular Interactions

In order to visualize the intramolecular NCI occurring in 2ClPA, a graphical method related to the representation of the electron density properties proposed by Johnson^25^ was applied. The gradient’s iso-surfaces of the electron density are colored according to the corresponding values of sign­(λ_2_)­ρ. A negative sign­(λ_2_)­ρ (corresponding to blue regions) suggests stronger attractive interactions, while green regions indicate weaker ones. On the contrary, positive values of sign­(λ_2_)­ρ and orange or red regions indicate weak or stronger repulsive interaction, respectively.

From the plots reported in Figure 4, it is seen that the two most stable conformers possess a Z orientation of the carboxylic group. Specifically, 2ClPA-I exhibits a weaker interaction between the terminal methyl hydrogens and the carbonyl oxygen, while 2ClPA-II shows a weaker interaction between the terminal methyl hydrogens and the acidic oxygen. Since the methyl and carboxyl groups are far away from the chlorine nucleus, the weak interaction between them does not have a significant effect on the electric field gradient at the quadrupole site, which is consistent with the smaller η value obtained.

**Figure 4:**
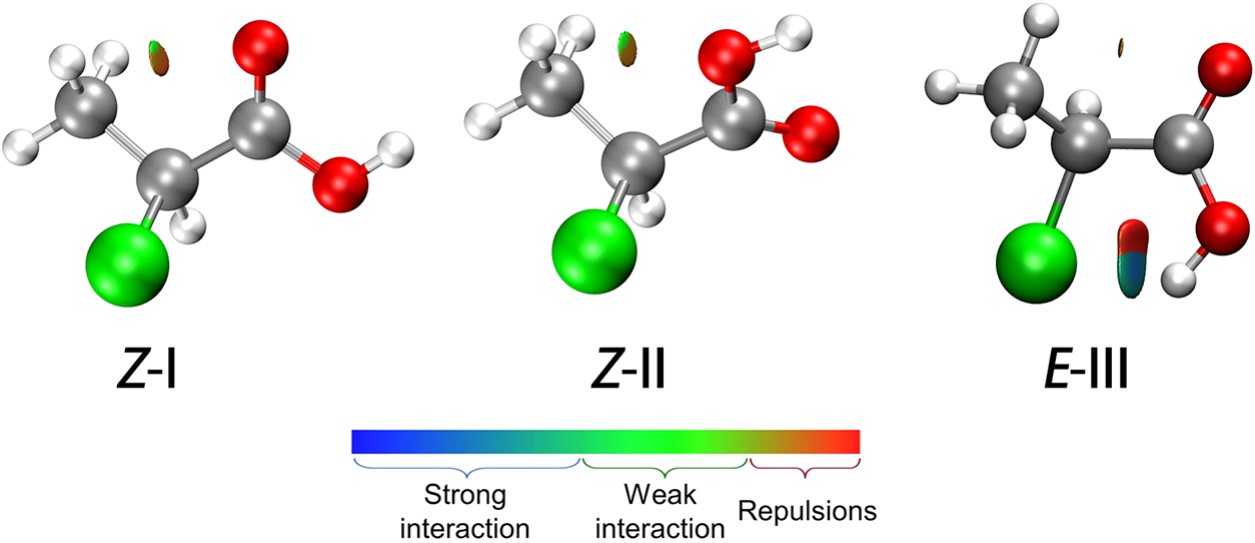
NCI plots from the B3LYP-D3­(BJ)/def2-TZVP optimization outputs for the three conformers of 2ClPA. Gradient iso-surfaces according to the values of the sign­(λ_2_)­ρ (−0.05, 0.05 a.u.). Color coding is blue (stronger attractive interactions), green (weak interactions) and orange-red (repulsive interaction).

For the *E*-III species, the shapes of the iso-surfaces imply that while CH···O=C interaction is still in place, a stronger Cl···HO interaction plays a crucial role in the stability of this conformation. A bond critical point was also found between the Cl and the HO groups, where the all-electron densities of 2.41 × 10^–2^ a.u. (B3LYP-D3­(BJ)/def2-TZVP) or 2.59 × 10^–2^ a.u. (MP2/aug-cc-pVTZ) where found.

The asymmetry of intramolecular interactions near the chlorine nucleus causes the asymmetry of the electric field gradient, being the origin of the significantly larger η value for this conformer.

### Conformational Equilibria

An overall discussion of the conformational space of propionic acid and its chlorine substituted propionic acids can now be attempted based on the experimental observations and the structurally relaxed energy scans reported in Figure 5 for propionic acid and its monochlorinated forms. A first inspection of the PESs reveals that the delicate interplay between attractive and repulsive forces, which governs both the conformer stability and the barrier heights, markedly alters the conformational landscape depending on the position of substitution.

**Figure 5:**
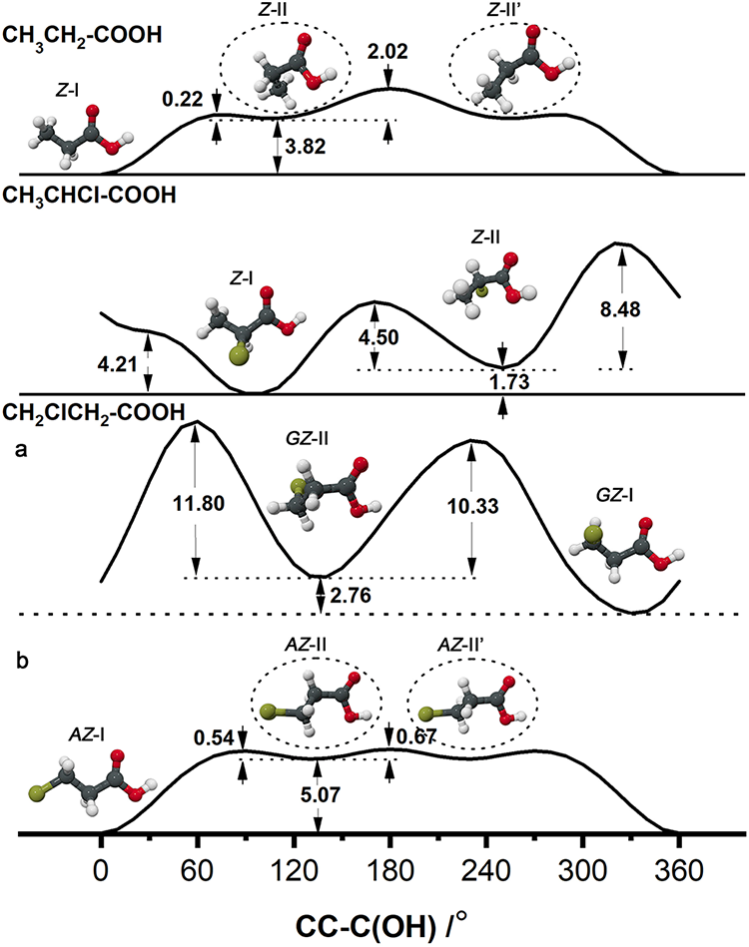
Potential energy diagrams (reported values in kJ mol^–1^) of the carboxyl group torsion in propionic acid and chlorine-substituted derivatives. Structurally relaxed scans along the dihedral angle CC-C­(OH) were performed at the B3LYP-D3­(BJ)/def2-TZVP level using 10° steps on the full cycle of rotation. Diagrams (a) and (b) correspond to the *gauche* and *anti* arrangements of the ClC–CC dihedral angle in 3ClPA, respectively. All conformations except those with dashed contours have been experimentally observed.^37,38,12^

As can be seen in Figure 5, the *Z-*II and *Z*-II′ conformations of propionic acid have not been observed in the gas phase due to the higher relative conformational energy (3.82 kJ mol^–1^) and lower interconversion barrier from *Z*-II to *Z*-I (0.22 kJ mol^–1^). However, when chlorine substitutes hydrogen at the α and β positions of propionic acid, a weak interaction forms between the chlorine and carboxyl groups, frustrating the rotation of the carboxyl group. Correspondingly, the barrier for conformational conversion from *Z*-II to *Z*-I becomes significantly higher (4.5 kJ mol^–1^ for 2ClPA and 10.33 kJ mol^–1^ for 3ClPA). It should be noted that the four conformations of 3ClPA involve two types of *Z-*I and *Z*-II conformational conversions: (a) *Z-*I to *Z*-II conversion with the ClC–CC angle in the *gauche* position and (b) the *Z-*I and *Z*-II conversion where the ClC–CC dihedral angle is in the *anti* orientation. It can be observed that due to the influence of chlorine, when the chlorine atom is close to the carboxyl group (a), *GZ-*I and *GZ*-II are trapped in a potential well with a high barrier (10.33 kJ mol^–1^), resulting in both *GZ*-I and *GZ*-II (*G*′*G*′*Z-*III and *G*′*AZ*-II in ref 11 where a three-letter notation was used) conformers being observed in the gas phase. However, when the chlorine atom is far from the carboxyl group, the energy curve (b) is similar to that of propionic acid, with the same low conversion barrier (0.54 kJ mol^–1^), and *AZ*-II (*AGZ*-IV in ref 11) was not observed in the gas phase. In our previous work on 3ClPA and this work on 2ClPA, we were able to experimentally characterize the structure of three of the four most stable conformations of 3ClPA and the two previously undetected conformations of 2ClPA (including the E-COOH rotamer), testing and verifying the accuracy of the theoretical calculations in describing the conformational behavior of complex molecular systems in isolated conditions.

## Conclusions

The rotational spectrum of 2-chloropropionic acid has been reinvestigated using millimeter-wave Stark-modulated absorption and centimeter-wave chirp-polarization Fourier transform microwave spectroscopy under isolated conditions in supersonic expansions. Not only the global minimum (*Z*-I) reported previously, but also two higher-energy conformers have been identified: the *Z*-II form, characterized by a *gauche* arrangement of the methyl group relative to the carbonyl, and the *E*-III form, stabilized by an intramolecular Cl···HO hydrogen bond.

Comparison between experimental and theoretical values confirms that B3LYP-D3­(BJ)/def2-TZVP tends to underestimate, whereas MP2/aug-cc-pVTZ slightly overestimates the rotational constants. Both approaches reproduce the molecular geometries within 1–2%, while the best agreement for the nuclear quadrupole coupling constants is obtained using the relativistically corrected MP2//DK method, with deviations below 5%. The diagonalization of the experimental NQCC tensor confirms that the principal *z*-axis of the electric field gradient coincides with the C–Cl bond. The larger asymmetry parameter η observed for the *E*-III conformer constitutes direct experimental evidence of the Cl···HO interaction on the local electronic environment of the chlorine nucleus.

A comparative analysis of the potential energy surfaces of propionic acid and its chlorinated derivatives highlights the key role of the chlorine atom in modulating the conformational flexibility. When chlorine is located close to the carboxyl group, the interaction between them significantly increases the interconversion barrier, stabilizing multiple conformers in the gas phase. Conversely, when chlorine is positioned farther from the COOH group, the potential surface resembles that of unsubstituted propionic acid, and higher-energy forms undergo relaxation during expansion.

Overall, this work completes the conformational characterization of 2ClPA in the gas phase and demonstrates how halogen substitution profoundly alters the electronic and structural landscape of simple carboxylic acids. The results underline the capability of rotational spectroscopy to detect subtle intramolecular interactions and to benchmark quantum-chemical methods for halogenated organic systems.

## Supplementary Material



## Data Availability

The computational data supporting the findings of this study are openly available in the University of Bologna repository at: 10.6092/unibo/amsacta/8723.

## Notes

Tables of measured transition lines for all observed conformers of 2ClPA (Tables S1–S6) (PDF)
