# Supplementary material for: Rotational Detection of the Missing Conformers of 2‑Chloropropionic Acid
Source: J Phys Chem A. 2026 Jan 15;130(4):836–44. doi: 10.1021/acs.jpca.5c07699 (PMC12862800; doi:10.1021/acs.jpca.5c07699)
Supplement: Supplementary file 1 [file jp5c07699_si_001.pdf]

Rotational detection of the missing conformers of 2-chloropropionic acid

*Fufei Sun, Assimo Maris, Luca Evangelisti, Wentao Song and Sonia Melandri**

Department of Chemistry "Giacomo Ciamician", University of Bologna, Via Gobetti 85, 40129 Bologna, Italy, sonia.melandri@unibo.it

J. Ricardo Morán, Camilla Calabrese, Alberto Lesarri*

Departamento de Química Física y Química Inorgánica, Facultad de Ciencias – I.U. CINQUIMA, Paseo de Belén, 7, 47011 Valladolid, Spain

Jens-Uwe Grabow

Institut für Physikalische Chemie und Elektrochemie, Gottfried-Wilhelm-Leibniz-Universität Hannover, Callinstrasse 3A, 30167 Hannover, Germany

Index

Table S1: Measured transition lines for ^{35}Cl-I of 2ClPA. Pg.3-8

Table S2: Measured transition lines for ^{37}Cl-I of 2ClPA. Pg. 9-11

Table S3: Measured transition lines for ^{35}Cl-II of 2ClPA. Pg. 12-14

Table S4: Measured transition lines for ^{37}Cl-II of 2ClPA. Pg. 15-16

Table S5: Measured transition lines for ^{35}Cl-III of 2ClPA. Pg. 17-18

Table S6: Measured transition lines for ^{37}Cl-III of 2ClPA. Pg. 19-20

References Pg.21

Table S1. Measured transition lines for ^{35}Cl-I of 2ClPA: (column 1-4) upper state rotational quantum numbers, (column 5-8) lower state rotational quantum numbers, observed transition frequencies $\nu_{(exp)}$ (MHz), discrepancies between observed and calculated frequencies $\Delta\nu$ (MHz), measurements' uncertainties (*Error* (MHz)). Hyperfine components are labelled as $F' \leftarrow F''$ where $F = I + J$ and half integer spins are rounded up to the next integer.

J'	K_a'	K_c'	F'	J''	K_a''	K_c''	F''	$\nu_{(exp)}$	$\Delta\nu$	*Error*
1	0	1	2	0	0	0	2	4075.96	0.00	0.01
1	0	1	3	0	0	0	2	4080.45	0.00	0.01
1	0	1	1	0	0	0	2	4083.89	0.00	0.01
2	1	2	3	1	1	1	2	7544.30	0.00	0.01
2	1	2	3	1	1	1	3	7545.30	0.00	0.01
2	1	2	2	1	1	1	2	7546.78	0.00	0.01
2	1	2	4	1	1	1	3	7548.73	0.00	0.01
2	1	2	1	1	1	1	1	7551.98	0.00	0.01
2	1	2	1	1	1	1	2	7550.17	0.00	0.01
4	1	3	6	4	1	4	6	5957.49	0.00	0.01
4	1	3	3	4	1	4	3	5956.66	-0.01	0.01
4	1	3	4	4	1	4	4	5959.01	0.00	0.01
4	1	3	5	4	1	4	5	5959.84	0.00	0.01
1	1	1	1	0	0	0	2	5628.33	0.00	0.01
1	1	1	3	0	0	0	2	5629.14	0.00	0.01
1	1	1	2	0	0	0	2	5630.13	0.00	0.01
3	1	2	4	3	0	3	4	4276.44	0.00	0.01
3	1	2	3	3	0	3	3	4275.15	0.00	0.01
3	1	2	5	3	0	3	5	4273.70	0.00	0.01
3	1	2	2	3	0	3	2	4272.44	0.00	0.01
3	2	2	2	3	1	3	2	7481.23	0.01	0.01
3	2	2	5	3	1	3	5	7483.38	0.01	0.01
3	2	2	3	3	1	3	3	7485.80	0.00	0.01
3	2	2	4	3	1	3	3	7485.80	0.00	0.01
3	2	2	4	3	1	3	4	7487.96	0.00	0.01
4	2	2	6	4	1	3	6	4702.86	0.00	0.01
4	2	2	5	4	1	3	5	4704.57	0.00	0.01
4	2	2	4	4	1	3	4	4703.96	-0.01	0.01
4	2	2	3	4	1	3	3	4702.25	0.00	0.01
1	1	0	1	0	0	0	2	6237.58	0.00	0.01
1	1	0	3	0	0	0	2	6240.21	0.00	0.01
1	1	0	2	0	0	0	2	6243.70	0.00	0.01
2	0	2	3	1	1	0	2	5843.46	0.00	0.01
2	0	2	2	1	1	0	2	5846.80	0.00	0.01

2	0	2	3	1	1	0	3	5846.95	0.00	0.01
2	0	2	4	1	1	0	3	5851.59	0.00	0.01
2	0	2	2	1	1	0	1	5852.92	0.00	0.01
2	0	2	1	1	1	0	1	5857.53	0.00	0.01
2	0	2	1	1	1	0	2	5851.41	0.00	0.01
3	1	3	4	2	2	1	3	4747.42	0.00	0.01
3	1	3	3	2	2	1	3	4749.57	0.00	0.01
3	1	3	4	2	2	1	4	4751.89	0.01	0.01
3	1	3	3	2	2	1	2	4752.76	0.00	0.01
3	1	3	5	2	2	1	4	4756.46	0.00	0.01
3	1	3	2	2	2	1	2	4757.35	0.00	0.01
3	1	3	2	2	2	1	1	4761.72	0.00	0.01
4	1	4	5	3	2	2	4	7367.32	0.00	0.01
4	1	4	4	3	2	2	3	7369.11	0.00	0.01
4	1	4	6	3	2	2	5	7372.42	0.00	0.01
4	1	4	3	3	2	2	2	7374.22	0.00	0.01
4	2	3	5	3	3	1	4	6834.78	0.00	0.01
4	2	3	4	3	3	1	3	6838.05	0.00	0.01
4	2	3	6	3	3	1	5	6842.20	0.00	0.01
4	2	3	3	3	3	1	2	6845.44	-0.01	0.01
2	2	0	4	2	1	1	4	4795.92	0.00	0.01
2	2	0	3	2	1	1	3	4801.58	0.00	0.01
2	2	0	1	2	1	1	2	4792.94	-0.01	0.01
2	2	0	2	2	1	1	3	4798.23	0.00	0.01
2	2	0	2	2	1	1	2	4797.52	0.00	0.01
2	2	0	1	2	1	2	1	6624.18	0.00	0.01
2	2	0	1	2	1	2	2	6627.56	0.00	0.01
2	2	0	2	2	1	2	1	6628.75	0.00	0.01
2	2	0	4	2	1	2	4	6629.85	0.00	0.01
2	2	0	2	2	1	2	2	6632.13	0.00	0.01
2	2	0	4	2	1	2	3	6633.29	0.00	0.01
2	2	0	3	2	1	2	2	6635.48	0.00	0.01
2	2	0	3	2	1	2	3	6637.96	0.00	0.01
2	2	1	1	2	1	1	1	4643.94	0.00	0.01
2	2	1	1	2	1	1	2	4644.96	0.00	0.01
2	2	1	4	2	1	1	4	4647.79	0.00	0.01
2	2	1	2	2	1	1	1	4648.31	0.00	0.01
2	2	1	4	2	1	1	3	4648.78	0.00	0.01
2	2	1	2	2	1	1	2	4649.33	0.00	0.01
2	2	1	2	2	1	1	3	4650.04	0.00	0.01
2	2	1	3	2	1	1	4	4652.24	0.00	0.01
2	2	1	3	2	1	1	2	4652.52	0.00	0.01
2	2	1	3	2	1	1	3	4653.23	0.00	0.01
3	2	2	2	3	1	2	2	3837.17	0.00	0.01

3	2	2	3	3	1	2	2	3837.17	0.00	0.01
3	2	2	5	3	1	2	5	3838.21	0.00	0.01
3	2	2	3	3	1	2	3	3839.37	0.00	0.01
3	2	2	4	3	1	2	3	3839.37	0.00	0.01
3	2	2	4	3	1	2	4	3840.38	0.00	0.01
4	2	3	3	4	1	3	3	2871.48	0.00	0.01
4	2	3	6	4	1	3	6	2871.82	0.00	0.01
4	2	3	4	4	1	3	4	2872.43	0.00	0.01
4	2	3	5	4	1	3	5	2872.76	0.00	0.01
4	3	2	3	4	2	2	3	7884.43	0.00	0.01
4	3	2	6	4	2	2	6	7885.41	0.00	0.01
4	3	2	4	4	2	2	4	7887.19	0.00	0.01
4	3	2	5	4	2	2	5	7888.13	0.00	0.01
5	3	3	5	5	2	3	5	6739.94	0.00	0.01
5	3	3	6	5	2	3	6	6740.37	0.00	0.01
6	3	4	5	6	2	4	5	5346.87	0.00	0.01
6	3	4	8	6	2	4	8	5347.06	0.00	0.01
6	3	4	6	6	2	4	6	5347.69	0.00	0.01
6	3	4	7	6	2	4	7	5347.89	0.00	0.01
7	3	5	9	7	2	5	9	3873.18	0.00	0.01
7	3	5	8	7	2	5	8	3873.59	-0.01	0.01
2	0	2	2	1	0	1	1	8006.612	-0.001	0.005[a]
2	0	2	3	1	0	1	2	8011.197	0.000	0.005
2	0	2	4	1	0	1	3	8011.345	0.001	0.005
2	0	2	2	1	0	1	2	8014.543	0.002	0.005
2	1	1	2	1	0	1	1	10927.648	-0.001	0.005
2	1	1	1	1	0	1	1	10928.665	0.000	0.005
2	1	1	3	1	0	1	3	10930.374	0.000	0.005
2	1	1	4	1	0	1	3	10931.364	0.001	0.005
2	1	1	3	1	0	1	2	10934.865	0.002	0.005
2	1	1	2	1	0	1	2	10935.576	-0.001	0.005
2	1	1	1	1	0	1	2	10936.594	0.002	0.005
3	1	2	2	2	0	2	1	15967.157	-0.001	0.005
3	1	2	5	2	0	2	4	15969.432	0.000	0.005
3	1	2	3	2	0	2	2	15969.568	0.000	0.005
3	1	2	4	2	0	2	3	15971.897	0.001	0.005
4	1	3	5	3	2	1	4	12635.477	0.001	0.005
4	1	3	6	3	2	1	5	12638.729	-0.003	0.005
2	2	0	2	1	1	0	2	13565.356	0.000	0.005
2	2	0	1	1	1	0	1	13566.905	0.002	0.005
2	2	0	4	1	1	0	3	13567.530	0.005	0.005
2	2	0	3	1	1	0	2	13568.706	0.003	0.005
2	2	0	2	1	1	0	1	13571.474	-0.001	0.005
2	2	0	3	1	1	0	3	13572.194	-0.001	0.005

2	2	1	4	1	1	1	3	14030.460	0.002	0.005
3	2	1	2	3	1	3	2	8172.176	-0.002	0.005
3	2	1	5	3	1	3	5	8174.551	0.001	0.005
3	2	1	3	3	1	3	3	8177.251	0.002	0.005
3	2	1	3	3	1	3	4	8179.393	-0.006	0.005
3	2	1	4	3	1	3	4	8179.637	-0.001	0.005
11	4	8	10	10	3	8	9	61760.28	0.02	0.05
			13				12	61760.28	0.02	0.05
11	4	8	11	10	3	8	10	61761.05	0.02	0.05
			12				11	61761.05	0.02	0.05
12	4	8	11	11	3	8	10	63106.93	0.00	0.05
			14				13	63106.93	0.00	0.05
12	4	8	12	11	3	8	11	63107.35	-0.01	0.05
			13				12	63107.35	-0.01	0.05
12	4	9	11	11	3	9	10	67234.33	0.01	0.05
			14				13	67234.33	0.01	0.05
12	4	9	12	11	3	9	11	67235.07	0.00	0.05
			13				12	67235.07	0.00	0.05
13	4	9	12	12	3	9	11	69175.53	-0.01	0.05
			15				14	69175.53	-0.01	0.05
13	4	9	13	12	3	9	12	69176.01	-0.01	0.05
			14				13	69176.01	-0.01	0.05
13	4	10	12	12	3	10	11	72858.07	-0.01	0.05
			15				14	72858.07	-0.01	0.05
13	4	10	13	12	3	10	12	72858.79	-0.02	0.05
			14				13	72858.79	-0.02	0.05
11	5	6	13	10	4	6	12	60007.94	-0.01	0.05
			10				9	60007.94	-0.01	0.05
11	5	6	12	10	4	6	11	60008.32	-0.01	0.05
			11				10	60008.32	-0.01	0.05
11	5	7	10	10	4	7	9	62796.99	0.03	0.05
			13				12	62796.99	0.03	0.05
11	5	7	11	10	4	7	10	62797.61	0.03	0.05
			12				11	62797.61	0.03	0.05
12	5	7	14	11	4	7	13	63795.27	-0.01	0.05
			11				10	63795.27	-0.01	0.05
12	5	7	13	11	4	7	12	63795.6	0.00	0.05
			12				11	63795.6	0.00	0.05
12	5	8	11	11	4	8	10	67623.3	0.01	0.05
			14				13	67623.3	0.01	0.05
12	5	8	12	11	4	8	11	67623.89	0.00	0.05
			13				12	67623.89	0.00	0.05
13	5	8	12	12	4	8	11	68117.25	-0.03	0.05

			15				14	68117.25	-0.03	0.05
13	5	8	13	12	4	8	12	68117.57	-0.01	0.05
			14				13	68117.57	-0.01	0.05
13	5	9	12	12	4	9	11	72701.8	0.02	0.05
			15				14	72701.8	0.02	0.05
13	5	9	13	12	4	9	12	72702.36	0.00	0.05
			14				13	72702.36	0.00	0.05
10	6	4	11	9	5	4	10	61157.29	0.00	0.05
			10				9	61157.29	0.00	0.05
10	6	4	12	9	5	4	11	61156.72	0.03	0.05
			9				8	61156.72	0.03	0.05
10	6	5	12	9	5	5	11	61393.22	0.02	0.05
			9				8	61393.22	0.02	0.05
10	6	5	11	9	5	5	10	61393.84	0.01	0.05
			10				9	61393.84	0.01	0.05
9	7	2	11	8	6	2	10	60942.67	0.04	0.05
			8				7	60942.67	0.04	0.05
9	7	2	10	8	6	2	9	60943.22	0.01	0.05
			9				8	60943.22	0.01	0.05
9	7	3	11	8	6	3	10	60945.55	0.02	0.05
			8				7	60945.55	0.02	0.05
9	7	3	10	8	6	3	9	60946.11	0.01	0.05
			9				8	60946.11	0.01	0.05
10	7	3	12	9	6	3	11	65038.29	0.01	0.05
			9				8	65038.29	0.01	0.05
10	7	3	11	9	6	3	10	65038.85	-0.01	0.05
			10				9	65038.85	-0.01	0.05
10	7	4	12	9	6	4	11	65051.94	-0.01	0.05
			9				8	65051.94	-0.01	0.05
10	7	4	11	9	6	4	10	65052.53	-0.01	0.05
			10				9	65052.53	-0.01	0.05
8	8	0	10	7	7	0	9	60491.68	0.01	0.05
			9				8	60491.68	0.01	0.05
			7				6	60491.68	0.01	0.05
			8				7	60491.68	0.01	0.05
8	8	1	10	7	7	1	9	60491.68	0.01	0.05
			9				8	60491.68	0.01	0.05
			7				6	60491.68	0.01	0.05
			8				7	60491.68	0.01	0.05
9	8	1	11	8	7	1	10	64617.57	0.03	0.05
			8				7	64617.57	0.03	0.05
9	8	1	10	8	7	1	9	64617.96	0.02	0.05
			9				8	64617.96	0.02	0.05
9	8	2	11	8	7	2	10	64617.57	-0.03	0.05

			8				7	64617.57	-0.03	0.05
9	8	2	10	8	7	2	9	64617.96	-0.03	0.05
			9				8	64617.96	-0.03	0.05
10	8	2	12	9	7	2	11	68737.37	0.03	0.05
			9				8	68737.37	0.03	0.05
10	8	2	11	9	7	2	10	68737.79	0.01	0.05
			10				9	68737.79	0.01	0.05
			12				11	68737.79	0.01	0.05
			9				8	68737.79	0.01	0.05
10	8	3	11	9	7	3	10	68738.2	-0.03	0.05
			10				9	68738.2	-0.03	0.05
9	9	0	11	8	8	0	10	68281.05	-0.02	0.05
			10				9	68281.05	-0.02	0.05
			8				7	68281.05	-0.02	0.05
			9				8	68281.05	-0.02	0.05
9	9	1	11	8	8	1	10	68281.05	-0.02	0.05
			10				9	68281.05	-0.02	0.05
			8				7	68281.05	-0.02	0.05
			9				8	68281.05	-0.02	0.05
10	10	1	12	9	9	1	11	76070.11	-0.02	0.05
			9				8	76070.11	-0.02	0.05
			11				10	76070.11	-0.02	0.05
			10				9	76070.11	-0.02	0.05

[a] Experimental data with measurement uncertainties of 0.005 and 0.05 are taken from Ref. 1.

Table S2. Measured transition lines for ^{37}Cl-I of 2ClPA: (column 1-4) upper state rotational quantum numbers, (column 5-8) lower state rotational quantum numbers, observed transition frequencies $\nu_{(exp)}$ (MHz), discrepancies between observed and calculated frequencies $\Delta\nu$ (MHz), measurements' uncertainties (*Error* (MHz)). Hyperfine components are labelled as $F' \leftarrow F''$ where $F = I + J$ and half integer spins are rounded up to the next integer.

J'	K_a'	K_c'	F'	J''	K_a''	K_c''	F''	$\nu_{(exp)}$	$\Delta\nu$	*Error*
1	1	0	1	0	0	0	2	6161.61	0.00	0.01
1	1	0	3	0	0	0	2	6163.80	0.00	0.01
1	1	0	2	0	0	0	2	6166.65	0.00	0.01
3	1	3	5	2	2	1	4	4533.16	0.01	0.01
3	1	3	4	2	2	1	3	4525.39	0.00	0.01
3	1	3	2	2	2	1	1	4537.73	0.01	0.01
4	1	4	5	3	2	2	4	7115.53	0.00	0.01
4	1	4	4	3	2	2	3	7117.02	0.00	0.01
4	1	4	6	3	2	2	5	7119.79	0.00	0.01
4	1	4	3	3	2	2	2	7121.29	0.00	0.01
2	2	0	2	2	1	2	1	6632.92	0.00	0.01
2	2	0	1	2	1	2	2	6631.71	0.00	0.01
2	2	0	1	2	1	2	1	6628.92	0.00	0.01
2	2	0	2	2	1	2	2	6635.71	0.00	0.01
2	2	0	4	2	1	2	3	6636.58	0.00	0.01
2	2	0	3	2	1	2	2	6638.61	0.00	0.01
2	2	0	4	2	1	2	4	6633.76	0.00	0.01
2	2	0	3	2	1	2	3	6640.64	0.00	0.01
2	2	1	1	2	1	1	2	4714.86	0.00	0.01
2	2	1	1	2	1	1	1	4713.75	0.00	0.01
2	2	1	2	2	1	1	1	4717.63	0.00	0.01
2	2	1	4	2	1	1	3	4718.41	0.00	0.01
2	2	1	2	2	1	1	2	4718.74	0.00	0.01
2	2	1	2	2	1	1	3	4719.53	0.00	0.01
2	2	1	3	2	1	1	4	4721.24	-0.01	0.01
2	2	1	3	2	1	1	2	4721.56	0.00	0.01
2	2	1	4	2	1	1	4	4717.32	0.00	0.01
2	2	1	3	2	1	1	3	4722.34	0.00	0.01
3	2	1	4	3	1	2	4	4579.08	0.01	0.01
3	2	2	5	3	1	2	5	3928.04	0.00	0.01
3	2	2	3	3	1	2	3	3929.17	0.00	0.01
3	2	2	4	3	1	2	4	3930.16	0.00	0.01
4	2	3	3	4	1	3	3	2979.06	0.00	0.01
4	2	3	6	4	1	3	6	2979.41	0.00	0.01

4	2	3	4	4	1	3	4	2980.06	0.00	0.01
4	2	3	5	4	1	3	5	2980.41	0.00	0.01
5	3	3	7	5	2	3	7	6924.19	0.00	0.01
5	3	3	4	5	2	3	4	6923.77	0.00	0.01
5	3	3	5	5	2	3	5	6925.28	0.00	0.01
5	3	3	6	5	2	3	6	6925.70	0.00	0.01
6	3	4	5	6	2	4	5	5566.94	0.00	0.01
6	3	4	8	6	2	4	8	5567.15	0.00	0.01
6	3	4	6	6	2	4	6	5567.82	0.00	0.01
6	3	4	7	6	2	4	7	5568.03	-0.01	0.01
1	0	1	3	0	0	0	2	3999.38	0.00	0.01
1	0	1	1	0	0	0	2	4002.45	0.00	0.01
1	0	1	2	0	0	0	2	3995.42	0.00	0.01
2	0	2	3	1	0	1	3	7855.05	0.00	0.01
2	0	2	2	1	0	1	1	7854.89	0.00	0.01
2	0	2	1	1	0	1	1	7858.91	0.00	0.01
2	0	2	2	1	0	1	2	7861.91	0.00	0.01
2	0	2	3	1	1	0	2	5687.78	0.00	0.01
2	0	2	1	1	1	0	1	5699.74	0.00	0.01
2	0	2	2	1	1	0	1	5695.72	0.00	0.01
2	0	2	4	1	1	0	3	5694.67	0.00	0.01
2	0	2	2	1	1	0	2	5690.67	-0.01	0.01
2	0	2	3	1	1	0	3	5690.64	0.01	0.01
2	1	2	3	1	1	1	2	7401.42	0.00	0.01
2	1	2	3	1	1	1	3	7402.52	0.00	0.01
2	1	2	2	1	1	1	2	7403.45	0.00	0.01
2	1	2	4	1	1	1	3	7405.34	0.00	0.01
2	1	2	1	1	1	1	2	7406.23	-0.01	0.01
2	1	2	1	1	1	1	1	7408.23	0.00	0.01
2	0	2	4	1	1	1	3	6287.18	0.00	0.01
2	0	2	3	1	1	1	2	6282.05	0.01	0.01
2	0	2	2	1	1	1	2	6284.94	-0.01	0.01
2	1	1	2	1	0	1	1	10751.919	0.001	0.005[a]
2	1	1	1	1	0	1	1	10753.030	0.000	0.005
2	1	1	3	1	0	1	3	10754.201	-0.002	0.005
2	1	1	4	1	0	1	3	10755.298	0.000	0.005
2	1	1	3	1	0	1	2	10758.157	0.000	0.005
2	1	1	2	1	0	1	2	10758.943	-0.001	0.005
2	1	1	1	1	0	1	2	10760.056	-0.002	0.005
2	2	0	2	1	1	0	2	13444.893	-0.001	0.005
2	2	0	1	1	1	0	1	13445.934	-0.001	0.005
2	2	0	4	1	1	0	3	13446.601	0.004	0.005
2	2	0	3	1	1	0	2	13447.800	0.001	0.005
2	2	0	2	1	1	0	1	13449.931	0.000	0.005

2	2	0	3	1	1	0	3	13450.656	0.001	0.005
3	1	2	2	2	0	2	1	15680.384	0.001	0.005
3	1	2	4	2	0	2	4	15680.147	0.000	0.005
3	1	2	5	2	0	2	4	15682.264	0.001	0.005
3	1	2	4	2	0	2	3	15684.183	0.001	0.005
8	8	1	10	7	7	1	9	60049.03	0.00	0.05
			9				8	60049.03	0.00	0.05
			7				6	60049.03	0.00	0.05
			8				7	60049.03	0.00	0.05
9	7	2	11	8	6	2	10	60386.03	0.01	0.05
			8				7	60386.03	0.01	0.05
9	7	2	10	8	6	2	9	60386.60	0.07	0.05
			9				8	60386.60	0.07	0.05
9	7	3	11	8	6	3	10	60388.34	-0.02	0.05
			8				7	60388.34	-0.02	0.05
9	7	3	10	8	6	3	9	60388.81	-0.06	0.05
			9				8	60388.81	-0.06	0.05
10	6	4	11	9	5	4	10	60513.38	0.00	0.05
			10				9	60513.38	0.00	0.05
10	6	4	12	9	5	4	11	60512.86	0.03	0.05
			9				8	60512.86	0.03	0.05
10	6	5	12	9	5	5	11	60714.00	-0.03	0.05
			9				8	60714.00	-0.03	0.05
10	6	5	11	9	5	5	10	60714.58	-0.01	0.05
			10				9	60714.58	-0.01	0.05

[a] Experimental data with measurement uncertainties of 0.005 and 0.05 are taken from Ref. 1.

Table S3. Measured transition lines for ^{35}Cl-II of 2ClPA: (column 1-4) upper state rotational quantum numbers, (column 5-8) lower state rotational quantum numbers, observed transition frequencies $\nu_{(exp)}$ (MHz), discrepancies between observed and calculated frequencies $\Delta\nu$ (MHz), measurements' uncertainties (*Error* (MHz)). Hyperfine components are labelled as $F' \leftarrow F''$ where $F = I + J$ and half integer spins are rounded up to the next integer.

J'	K_a'	K_c'	F'	J''	K_a''	K_c''	F''	$\nu_{(exp)}$	$\Delta\nu$	*Error*
11	5	7	10	10	4	6	9	62164.28	-0.04	0.05
11	5	7	13	10	4	6	12			
11	5	7	11	10	4	6	10			
11	5	7	12	10	4	6	11			
11	5	6	10	10	4	7	9	62351.92	0.01	0.05
11	5	6	13	10	4	7	12			
12	5	8	11	11	4	7	10	65959.50	-0.02	0.05
12	5	8	14	11	4	7	13			
12	5	8	12	11	4	7	11			
12	5	8	13	11	4	7	12			
12	5	7	11	11	4	8	10	66353.54	-0.05	0.05
12	5	7	14	11	4	8	13			
12	5	7	12	11	4	8	11	66354.35	0.03	0.05
12	5	7	13	11	4	8	12			
13	5	9	12	12	4	8	11	69641.09	0.03	0.05
13	5	9	15	12	4	8	14			
13	5	9	13	12	4	8	12			
13	5	9	14	12	4	8	13			
12	5	7	11	11	4	8	10	66353.61	0.02	0.05
12	5	7	14	11	4	8	13			
12	5	7	12	11	4	8	11	66354.34	0.02	0.05
12	5	7	13	11	4	8	12			
10	6	4	9	9	5	5	8	62522.72	-0.02	0.05
10	6	4	12	9	5	5	11			
10	6	4	10	9	5	5	9	62523.56	0.02	0.05
10	6	4	11	9	5	5	10			
11	6	6	10	10	5	5	9	66474.07	0.00	0.05
11	6	6	13	10	5	5	12			
11	6	6	11	10	5	5	10	66474.74	-0.02	0.05
11	6	6	12	10	5	5	11			
11	6	5	10	10	5	6	9	66482.18	-0.03	0.05
11	6	5	13	10	5	6	12			
11	6	5	11	10	5	6	10	66482.96	0.05	0.05

11	6	5	12	10	5	6	11			
12	6	7	11	11	5	6	10	70413.68	0.01	0.05
12	6	7	14	11	5	6	13			
12	6	7	12	11	5	6	11	70414.25	0.00	0.05
12	6	7	13	11	5	6	12			
12	6	6	11	11	5	7	10	70435.27	0.01	0.05
12	6	6	14	11	5	7	13			
12	6	6	12	11	5	7	11	70435.87	-0.02	0.05
12	6	6	13	11	5	7	12			
13	6	8	12	12	5	7	11	74331.20	0.00	0.05
13	6	8	15	12	5	7	14			
13	6	8	13	12	5	7	12			
13	6	8	14	12	5	7	13			
13	6	7	12	12	5	8	11	74382.88	-0.05	0.05
13	6	7	15	12	5	8	14			
13	6	7	13	12	5	8	12	74383.50	0.02	0.05
13	6	7	14	12	5	8	13			
9	7	3	8	8	6	2	7	62704.76	0.01	0.05
9	7	2	8	8	6	3	7			
9	7	3	11	8	6	2	10			
9	7	2	11	8	6	3	10			
9	7	3	9	8	6	2	8	62705.51	0.00	0.05
9	7	2	9	8	6	3	8			
9	7	3	10	8	6	2	9			
9	7	2	10	8	6	3	9			
10	7	4	9	9	6	3	8	66676.15	0.01	0.05
10	7	3	9	9	6	4	8			
10	7	4	12	9	6	3	11			
10	7	3	12	9	6	4	11			
10	7	4	10	9	6	3	9	66676.91	0.01	0.05
10	7	3	10	9	6	4	9			
10	7	4	11	9	6	3	10			
10	7	3	11	9	6	4	10			
11	7	5	10	10	6	4	9	70643.96	0.01	0.05
11	7	5	13	10	6	4	12			
11	7	4	10	10	6	5	9			
11	7	4	13	10	6	5	12			
11	7	5	11	10	6	4	10	70644.63	-0.04	0.05
11	7	5	12	10	6	4	11			
11	7	4	11	10	6	5	10			
11	7	4	12	10	6	5	11			
12	7	6	11	11	6	5	10	74606.46	0.03	0.05
12	7	6	14	11	6	5	13			
12	7	6	12	11	6	5	11	74607.12	0.02	0.05

12	7	5	11	11	6	6	10			
12	7	6	13	11	6	5	12			
12	7	5	14	11	6	6	13			
12	7	5	12	11	6	6	11	74607.73	-0.04	0.05
12	7	5	13	11	6	6	12			
8	8	0	10	7	7	1	9	62872.12	-0.04	0.05
8	8	1	10	7	7	0	9			
8	8	0	7	7	7	1	6			
8	8	1	7	7	7	0	6			
8	8	0	9	7	7	1	8			
8	8	1	9	7	7	0	8			
8	8	0	8	7	7	1	7			
8	8	1	8	7	7	0	7			
9	8	2	8	8	7	1	7	66847.04	0.04	0.05
9	8	1	8	8	7	2	7			
9	8	2	11	8	7	1	10			
9	8	1	11	8	7	2	10			
9	8	2	9	8	7	1	8	66847.53	0.00	0.05
9	8	1	9	8	7	2	8			
9	8	2	10	8	7	1	9			
9	8	1	10	8	7	2	9			
10	8	3	9	9	7	2	8	70820.96	0.03	0.05
10	8	2	9	9	7	3	8			
10	8	3	12	9	7	2	11			
10	8	2	12	9	7	3	11			
10	8	3	10	9	7	2	9	70821.55	-0.02	0.05
10	8	2	10	9	7	3	9			
10	8	3	11	9	7	2	10			
10	8	2	11	9	7	3	10			
11	8	4	10	10	7	3	9	74793.07	0.00	0.05
11	8	3	10	10	7	4	9			
11	8	4	13	10	7	3	12			
11	8	3	13	10	7	4	12			
11	8	4	11	10	7	3	10	74793.73	-0.01	0.05
11	8	3	11	10	7	4	10			
11	8	4	12	10	7	3	11			
11	8	3	12	10	7	4	11			

Table S4. Measured transition lines for ^{37}Cl-II of 2ClPA: (column 1-4) upper state rotational quantum numbers, (column 5-8) lower state rotational quantum numbers, observed transition frequencies $\nu_{(exp)}$ (MHz), discrepancies between observed and calculated frequencies $\Delta\nu$ (MHz), measurements' uncertainties (*Error* (MHz)). Hyperfine components are labelled as $F' \leftarrow F''$ where $F = I + J$ and half integer spins are rounded up to the next integer.

J'	K_a'	K_c'	F'	J''	K_a''	K_c''	F''	$\nu_{(exp)}$	$\Delta\nu$	*Error*
9	7	3	8	8	6	2	7	62166.58	0.03	0.05
9	7	2	8	8	6	3	7			
9	7	3	11	8	6	2	10			
9	7	2	11	8	6	3	10			
9	7	3	9	8	6	2	8	62167.23	0.00	0.05
9	7	2	9	8	6	3	8			
9	7	3	10	8	6	2	9			
9	7	2	10	8	6	3	9			
10	7	4	9	9	6	3	8	66059.29	0.04	0.05
10	7	3	9	9	6	4	8			
10	7	4	12	9	6	3	11			
10	7	3	12	9	6	4	11			
10	7	4	10	9	6	3	9			
10	7	3	10	9	6	4	9			
10	7	4	11	9	6	3	10			
10	7	3	11	9	6	4	10			
11	7	5	10	10	6	4	9	69947.79	-0.05	0.05
11	7	5	13	10	6	4	12			
11	7	4	10	10	6	5	9			
11	7	4	13	10	6	5	12			
11	7	5	11	10	6	4	10	69948.48	-0.01	0.05
11	7	5	12	10	6	4	11			
11	7	4	11	10	6	5	10			
11	7	4	12	10	6	5	11			
8	8	0	10	7	7	1	9	62439.83	0.01	0.05
8	8	1	10	7	7	0	9			
8	8	0	7	7	7	1	6			
8	8	1	7	7	7	0	6			
8	8	0	9	7	7	1	8			
8	8	1	9	7	7	0	8			
8	8	0	8	7	7	1	7			
8	8	1	8	7	7	0	7			
9	8	2	8	8	7	1	7	66335.75	-0.01	0.05

9	8	1	8	8	7	2	7			
9	8	2	11	8	7	1	10			
9	8	1	11	8	7	2	10			
9	8	2	9	8	7	1	8			
9	8	1	9	8	7	2	8			
9	8	2	10	8	7	1	9			
9	8	1	10	8	7	2	9			
10	8	3	9	9	7	2	8	70230.32	-0.01	0.05
10	8	2	9	9	7	3	8			
10	8	3	12	9	7	2	11			
10	8	2	12	9	7	3	11			
10	8	3	10	9	7	2	9	70230.88	-0.02	0.05
10	8	2	10	9	7	3	9			
10	8	3	11	9	7	2	10			
10	8	2	11	9	7	3	10			
10	6	5	9	9	5	4	8	61876.73	-0.01	0.05
10	6	5	12	9	5	4	11			
10	6	5	10	9	5	4	9	61877.48	0.02	0.05
10	6	5	11	9	5	4	10			
10	6	4	9	9	5	5	8	61879.18	0.03	0.05
10	6	4	12	9	5	5	11			

Table S5. Measured transition lines for ^{35}Cl-III of 2ClPA: (column 1-4) upper state rotational quantum numbers, (column 5-8) lower state rotational quantum numbers, observed transition frequencies $\nu_{(exp)}$ (MHz), discrepancies between observed and calculated frequencies $\Delta\nu$ (MHz), measurements' uncertainties (*Error* (MHz)). Hyperfine components are labelled as $F' \leftarrow F''$ where $F = I + J$ and half integer spins are rounded up to the next integer.

J'	K_a'	K_c'	F'	J''	K_a''	K_c''	F''	$\nu_{(exp)}$	$\Delta\nu$	*Error*
1	0	1	2	0	0	0	2	3917.95	0.01	0.01
1	0	1	3	0	0	0	2	3927.09	0.02	0.01
1	0	1	1	0	0	0	2	3934.29	0.00	0.01
2	0	2	3	1	0	1	2	7716.03	0.00	0.01
2	0	2	1	1	0	1	1	7716.65	0.00	0.01
2	0	2	4	1	0	1	3	7716.78	0.00	0.01
2	0	2	2	1	0	1	1	7706.78	0.00	0.01
2	0	2	3	1	0	1	3	7706.90	0.00	0.01
2	0	2	2	1	0	1	2	7723.12	0.01	0.01
2	0	2	1	1	0	1	2	7732.99	0.00	0.01
2	0	2	2	1	0	1	3	7713.99	0.00	0.01
2	1	2	4	1	1	1	3	7203.23	0.00	0.01
2	1	2	2	1	1	1	1	7201.32	-0.01	0.01
2	1	2	2	1	1	1	2	7200.15	0.00	0.01
2	1	2	3	1	1	1	2	7194.12	0.00	0.01
2	1	2	3	1	1	1	3	7194.81	0.00	0.01
2	1	2	1	1	1	1	1	7209.74	0.00	0.01
2	1	2	1	1	1	1	2	7208.57	0.00	0.01
2	1	2	2	1	1	1	3	7200.85	0.00	0.01
3	1	2	4	3	1	3	4	3886.07	0.01	0.01
3	1	2	5	3	1	3	5	3878.37	0.00	0.01
3	1	2	2	3	1	3	2	3874.77	0.00	0.01
3	1	2	3	3	1	3	3	3882.47	0.01	0.01
4	1	3	3	4	1	4	3	6370.50	0.00	0.01
4	1	3	6	4	1	4	6	6373.13	0.00	0.01
4	1	3	4	4	1	4	4	6377.97	-0.01	0.01
4	1	3	5	4	1	4	5	6380.60	0.00	0.01
5	2	3	7	5	2	4	7	3520.21	-0.01	0.01
5	2	3	6	5	2	4	6	3523.87	0.01	0.01
5	2	3	5	5	2	4	5	3522.83	0.00	0.01
2	0	2	2	1	1	1	2	5723.22	0.00	0.01
2	0	2	2	1	1	1	1	5724.40	0.01	0.01
2	0	2	3	1	1	1	2	5716.14	0.01	0.01
2	0	2	1	1	1	1	1	5734.27	0.01	0.01

2	0	2	4	1	1	1	3	5726.68	-0.01	0.01
2	0	2	3	1	1	1	3	5716.82	0.00	0.01
1	1	1	1	0	0	0	2	5916.68	0.01	0.01
1	1	1	3	0	0	0	2	5917.16	0.00	0.01
1	1	1	2	0	0	0	2	5917.85	0.01	0.01
2	1	1	4	2	0	2	4	3423.74	0.00	0.01
2	1	1	3	2	0	2	2	3425.88	0.00	0.01
2	1	1	2	2	0	2	2	3426.35	-0.01	0.01
2	1	1	1	2	0	2	2	3427.03	0.00	0.01
2	1	1	3	2	0	2	3	3432.97	0.00	0.01
2	1	1	4	2	0	2	3	3433.63	0.01	0.01
2	1	1	2	2	0	2	3	3433.44	0.00	0.01
3	1	2	3	2	2	1	2	6678.80	-0.02	0.01
3	1	2	5	2	2	1	4	6683.11	-0.02	0.01
3	1	2	5	3	0	3	5	4808.19	0.00	0.01
3	1	2	3	3	0	3	3	4812.29	0.00	0.01
3	1	2	4	3	0	3	4	4815.89	0.00	0.01
4	1	3	3	4	0	4	3	6875.86	0.00	0.01
4	1	3	6	4	0	4	6	6878.42	0.00	0.01
4	1	3	4	4	0	4	4	6883.16	0.01	0.01
4	1	3	5	4	0	4	5	6885.73	0.01	0.01
2	2	0	3	2	1	1	3	6118.34	-0.01	0.01
2	2	0	2	2	1	1	2	6110.78	-0.01	0.01
2	2	0	4	2	1	1	4	6107.78	-0.01	0.01
2	2	0	1	2	1	1	1	6100.25	-0.01	0.01
2	2	0	3	2	1	1	2	6117.87	0.00	0.01

Table S6. Measured transition lines for ^{37}Cl-III of 2ClPA: (column 1-4) upper state rotational quantum numbers, (column 5-8) lower state rotational quantum numbers, observed transition frequencies $\nu_{(exp)}$ (MHz), discrepancies between observed and calculated frequencies $\Delta\nu$ (MHz), measurements' uncertainties (*Error* (MHz)). Hyperfine components are labelled as $F' \leftarrow F''$ where $F = I + J$ and half integer spins are rounded up to the next integer.

J'	K_a'	K_c'	F'	J''	K_a''	K_c''	F''	$\nu_{(exp)}$	$\Delta\nu$	*Error*
1	0	1	3	0	0	0	2	3841.49	0.01	0.01
1	0	1	2	0	0	0	2	3834.10	0.01	0.01
1	0	1	1	0	0	0	2	3847.35	0.00	0.01
2	0	2	2	1	0	1	2	7562.95	0.00	0.01
2	0	2	4	1	0	1	3	7557.81	0.00	0.01
2	0	2	3	1	0	1	2	7557.26	0.00	0.01
2	0	2	1	1	0	1	1	7557.63	0.01	0.01
2	0	2	3	1	0	1	3	7549.87	0.00	0.01
2	0	2	2	1	0	1	1	7549.68	-0.01	0.01
2	1	2	4	1	1	1	3	7057.50	0.00	0.01
2	1	2	1	1	1	1	1	7062.81	-0.01	0.01
2	1	2	2	1	1	1	1	7056.19	-0.01	0.01
2	1	2	3	1	1	1	2	7050.12	0.00	0.01
2	1	2	3	1	1	1	3	7050.88	0.00	0.01
2	1	2	2	1	1	1	2	7054.87	0.00	0.01
2	1	2	1	1	1	1	2	7061.49	0.01	0.01
2	1	2	2	1	1	1	3	7055.62	0.00	0.01
3	1	2	5	3	1	3	5	3729.90	0.01	0.01
3	1	2	3	3	1	3	3	3732.99	0.00	0.01
3	1	2	4	3	1	3	4	3735.73	0.01	0.01
3	1	2	2	3	1	3	2	3727.18	0.02	0.01
1	1	1	3	0	0	0	2	5875.20	0.00	0.01
1	1	1	2	0	0	0	2	5875.95	-0.01	0.01
1	1	1	1	0	0	0	2	5874.63	0.00	0.01
2	1	1	4	2	0	2	4	3405.40	-0.01	0.01
2	1	1	3	2	0	2	3	3412.61	0.00	0.01
2	1	1	2	2	0	2	1	3399.51	0.00	0.01
2	1	1	1	2	0	2	1	3400.26	0.00	0.01
2	1	1	3	2	0	2	4	3404.65	-0.02	0.01
3	1	2	5	3	0	3	5	4719.70	0.00	0.01
3	1	2	4	3	0	3	4	4725.60	0.00	0.01
3	1	2	3	3	0	3	3	4722.84	0.00	0.01
3	1	2	2	3	0	3	2	4716.95	0.01	0.01
4	1	3	6	4	0	4	6	6691.73	-0.02	0.01

4	1	3	5	4	0	4	5	6697.31	0.00	0.01
4	1	3	4	4	0	4	4	6695.34	-0.01	0.01
3	2	1	5	3	1	2	5	5840.31	-0.01	0.01
3	2	1	4	3	1	2	4	5844.44	0.00	0.01
3	2	1	3	3	1	2	3	5842.52	0.00	0.01
3	2	1	2	3	1	2	2	5838.40	0.01	0.01
3	2	1	4	3	1	2	3	5843.15	0.01	0.01

References

1. Lesarri, A.; Grabow, J. U.; Caminati, W. Conformation of chiral molecules: The rotational spectrum of 2-chloropropionic acid. *Chem. Phys. Lett.* **2009**, *468* (1-3), 18-22.
